# Quercetin Ameliorates Neuropathic Pain after Brachial Plexus Avulsion *via* Suppressing Oxidative Damage through Inhibition of PKC/MAPK/NOX Pathway

**DOI:** 10.2174/1570159X21666230802144940

**Published:** 2023-09-01

**Authors:** Yanfeng Huang, Xie Zhang, Yidan Zou, Qiuju Yuan, Yan-Fang Xian, Zhi-Xiu Lin

**Affiliations:** 1 School of Chinese Medicine, Faculty of Medicine, The Chinese University of Hong Kong, Shatin, N.T., Hong Kong SAR, China;; 2 Research Center for Integrative Medicine of Guangzhou University of Chinese Medicine, Key Laboratory of Chinese Medicine Pathogenesis and Therapy Research, School of Basic Medical Sciences, Guangzhou University of Chinese Medicine, Guangzhou, Guangdong. P.R. China;; 3 Department of Medical Biotechnology, School of Basic Medical Sciences, Guangzhou University of Chinese Medicine, Guangzhou, Guangdong. P.R. China;; 4 Department of Anaesthesia and Intensive Care and Peter Hung Pain Research Institute, Faculty of Medicine, The Chinese University of Hong Kong, Shatin, N.T., Hong Kong SAR, China;; 5 Centre for Regenerative Medicine and Health, Hong Kong Institute of Science & Innovation, Chinese Academy of Sciences, Hong Kong Science Park, Shatin, N.T., Hong Kong SAR, China;; 6 Hong Kong Institute of Integrative Medicine, The Chinese University of Hong Kong, Hong Kong SAR, China

**Keywords:** Brachial plexus avulsion, neuropathic pain, quercetin, neuro-inflammatory infiltration, oxidative damage, PKC/MAPK/NOX pathway

## Abstract

**Background:**

Brachial plexus avulsion (BPA) animally involves the separation of spinal nerve roots themselves and the correlative spinal cord segment, leading to formidable neuropathic pain of the upper limb.

**Methods:**

The right seventh cervical (C7) ventral and dorsal roots were avulsed to establish a neuropathic pain model in rats. After operation, rats were treated with quercetin (QCN) by intragastric administration for 1 week. The effects of QCN were evaluated using mechanical allodynia tests and biochemical assay kits.

**Results:**

QCN treatment significantly attenuated the avulsion-provoked mechanical allodynia, elevated the levels of catalase (CAT), superoxide dismutase (SOD) and glutathione peroxidase (GPx) and total antioxidant capacity (TAC) in the C7 spinal dorsal horn. In addition, QCN administration inhibited the activations of macrophages, microglia and astrocytes in the C6 dorsal root ganglion (DRG) and C6-8 spinal dorsal horn, as well as attenuated the release of purinergic 2X (P2X) receptors in C6 DRG. The molecular mechanism underlying the above alterations was found to be related to the suppression of the PKC/MAPK/NOX signal pathway. To further study the anti-oxidative effects of QCN, we applied QCN on the H_2_O_2_-induced BV-2 cells *in vitro*, and the results attested that QCN significantly ameliorated the H_2_O_2_-induced ROS production in BV-2 cells, inhibited the H_2_O_2_-induced activation of PKC/MAPK/NOX pathway.

**Conclusion:**

Our study for the first time provided evidence that QCN was able to attenuate pain hypersensitivity following the C7 spinal root avulsion in rats, and the molecular mechanisms involve the reduction of both neuro-inflammatory infiltration and oxidative stress *via* suppression of P2X receptors and inhibition of the activation of PKC/MAPK/NOX pathway. The results indicate that QCN is a natural compound with great promise worthy of further development into a novel therapeutic method for the treatment of BPA-induced neuropathic pain.

## 1 INTRODUCTION

Brachial plexus avulsion (BPA, also named spinal root avulsion), characterized by pulling apart spinal nerves from their connection at the corresponding spinal cord segments, is most commonly caused by high kinetic trauma or complicated childbirth. Clinically, BPA is diagnosed in approximately 70% of grievous brachial plexus traction injuries [1]. Patients with this kind of lesion were usually subjected to neuropathic pain in the affected extremity [1, 2]. Neuropathic pain on account of nerve damage is a common pain situation, which is one of the intractable complications of BPA and is tightly involved in hyperalgesia (painful stimuli), allodynia (painful perception of non-noxious stimuli) and spontaneous pain of the affected limb [3, 4]. Almost 80% of patients with BPA develop chronic pain [5]. This type of pain typically manifests as hot-burning, tingling, pricking, pins-and-needles, sharp, shooting, squeezing, cold, electric or shock-like quality of pain [6]. Owing to the position of the brachial plexus, neuropathic pain caused by BPA implicates both the central nervous system (CNS) and peripheral nervous system (PNS) [7]. Hence, the mechanisms underlying the adult neuropathic pain after BPA are complex and concerned with structural and functional alterations by nociceptive pathways in both the CNS and PNS (the site of peripheral nerve injury, the spinal cord, as well as the dorsal root ganglion (DRG)) to generate the specific intensity and drawn-out time course of the pain [8-10].

During neuropathic pain induced by BPA, both spinal cord and nerve tissue are injured, releasing inflammatory and immunological substances such as the aggregation of glia cells (astrocyte and microglia) and macrophages, which can not only enable the excessive release of the extracellular adenosine triphosphate (ATP) but also activate the protein kinase C (PKC) pathway. Both ATP and its analogs can bring about the sensation of pain [11, 12]. ATP is involved in peripheral pain signals *via* purinergic 2X (P2X) receptors [13]. Several investigations have indicated that P2X receptors make a critical difference in supporting pain transmission at PNS and spinal sites for the reason that both sensory neurons and spinal cord dorsal horn neurons could be depolarized by ATP [14-17]. Furthermore, activation of the PKC pathway causes the phosphorylation of several intracellular targets recruiting diverse mitogen-activated protein kinase (MAPK) pathways, covering p38 MAPK, extracellular signal-regulated kinase 1/2 (ERK1/2) and C-jun N-terminal kinase (JNK). The activation of the PKC pathway plays a pivotal role as adjuster of nociceptive sensitivity in distinct models of pain and hyperalgesia to mechanical and thermal stimuli [18].

Additionally, oxidative stress is also closely concerned with the pathogenesis of neuropathic pain [19]. Increasing evidence suggests that reactive oxygen species (ROS), like hydrogen peroxide (H_2_O_2_) and superoxide (O_2_^−^) essentially accelerate nociceptive processing in the amygdala and conduce to promoting pain behavior during persistent pain [20]. Nicotinamide adenine dinucleotide phosphate (NADPH) oxidase (NOX) is one of the main sources of ROS in the nervous system [21, 22]. Previous studies demonstrated that PKC activation boosts the transposition of the cytosolic subunits to the plasma membrane, subsequently activates NOX, and eventually affects the frequency of action potentials in primary neurons, resulting in neuropathic pain [23-25].

The quality of life of BPA patients is possibly worsened by chronic and obstinate pain [26]. Pain treatment should be started as quickly as possible and should be aggressive. Neuropathic pain related to BPA is generally treated with medication first. However, the existing frontline pharmacological agents have a confined action and exist abuse liabilities, or unbearable adverse-effects [27]. Owing to both unique etiological and pathophysiological elements, pain after BPA is known to be arduous to treat, as it is resistive to most pain-soothing therapeutics [28]. Therefore, novel agents for the effective management of neuropathic pain with better safety profiles remain an unmet medical need.

Quercetin (QCN) is a ubiquitous polyphenolic compound detected in plentiful fruits, vegetables, tea and olive oil [29, 30]. It has been found that QCN processes remarkable anti-oxidative and anti-inflammatory properties [31, 32], as well as strong neuroprotective activity [33]. The analgesic effects of QCN have been disclosed in a variety of neuropathic pain animal models, such as paclitaxel-induced pain [34], spinal nerve ligation [35], spared nerve injury [36], chronic constriction injury of the sciatic nerve [37], and diabetic neuropathy [38].

Based on these promising discoveries, we put forward the following hypothesis: QCN administration is beneficial in ameliorating neuropathic pain in a rat model of BPA. Therefore, the aims of this study were (1) to investigate whether QCN treatment could alleviate neuropathic pain following unilateral C7 spinal root avulsion injury; and (2) to elucidate the underlying molecular mechanisms

## 2 MATERIALS AND METHODS

### 2.1 Animals

Adult female Sprague Dawley rats (weighing 180-220 g) were purchased from the Laboratory Animal Services Centre of The Chinese University of Hong Kong (CUHK) and were housed under standard environmental conditions at controlled temperature (22 ± 2°C), humidity (50 ± 10%), and light (12 h light/dark cycle) with free access to standard diet and water. All experimental protocols were approved by the Animal Experimentation Ethics Committee, CUHK (Ref. No.: 18/076/MIS-5-C).

### 2.2 Surgical Procedures

The C7 spinal root avulsion surgery was executed as a previous study [39]. Briefly, the rats were first anesthetized intraperitoneally with ketamine and xylazine (80 and 8 mg/kg, respectively). Under a stereomicroscope (magnification ×10), the right spine segments from the 6^th^ cervical (C6) to the 2^nd^ thoracic (T2) were carefully presented and identified depending on their location with respect to the long spine of T2. Then, a dorsal laminectomy was performed. The right side C7 dorsal and ventral roots were avulsed by pull with a fine glass hook, and the other parts of C7 were cut and removed, except a crack (around 5 mm) between the nerve roots and spinal cord was reserved to prevent any reconnection, after exposing the dura matter. Finally, the incision was stitched and the rats were put back in their individual cages to recover. Attention was paid to avoid any injury to the spinal cord. The right C7 spinal roots were merely exposed but not avulsed in the rats of the sham control group.

### 2.3 Treatment and Grouping

QCN was purchased from Sigma (St. Louis, MO, USA, lot numbers: Q4951, ≥ 95%). Rats were stochastically divided into five groups (n = 17): sham control group, vehicle control group, and QCN (25, 50 and 100 mg/kg) groups. Rats were intragastrically administered with QCN (25, 50 and 100 mg/kg) for 7 days, meanwhile, the rats in the sham control group and vehicle control group received an equal volume of vehicle (0.5% carboxymethyl cellulose sodium (CMC-Na)) for the same duration. QCN was suspended in 0.5% CMC-Na. Rats were treated daily with QCN or vehicle (0.5% CMC-Na) 2 h after surgery when the rats woke up. The doses of QCN were chosen according to the results of our previous study and other studies [34, 37, 40].

### 2.4 Behavioral Test for Mechanical Pain

After surgery, we performed the mechanical allodynia test which was taken as a behavioral sign of BPA-induced neuropathic pain as described by Hou and Xu [41]. Briefly, rats were first placed in a transparent Plexiglas chamber on the mesh stand that was placed at about 30 cm over the floor. Before the start of each test, rats acclimatized themselves to the trial environment for approximately 15 min until they quieted down and stood on all four feet. The mechanical hypersensitivity was assessed by the electronic Von Frey aesthesiometer (IITC Life Science, Woodland Hills, CA, USA) with a polypropylene rigid probe. The rigid probe was employed vertically to the middle plantar surface of the right forepaw of each rat with increasing stress. The paw withdrawal threshold (PWT, g) was automatically recorded using the aesthesiometer when the paw was withdrawn. A withdrawal reaction was thought to be available only if the anterior limb was totally moved away from the wire mesh platform. Three repetitive measurements were carried out at an interval of 30 seconds. The average PWT of the three repetitive measurements was calculated for each rat on days 0 (pre-injury) and 1, 3, 5, 7 post-injury. The data of day 0 (pre-injury) served as baseline. All the mechanical allodynia tests were conducted with two investigators who were unwitting of the grouping and treatment.

### 2.5 Behavioral Test for Thermal Hyperalgesia

Thermal hyperalgesia was assessed employing a plantar analgesia meter (Model 390G, IITC Life Science, Woodland Hills, CA, USA) as described by Hargreaves *et al.* [42]. The rats were put into a clear Plexiglas chamber (18 cm × 29 cm × 12.5 cm) on a glass plate positioned above a lightbox. After a 15-min period of habituation to their environment, radiant heat aimed at the middle plantar skin surface of the right forepaw was produced by turning on the lightbox. When the rat withdrew its forepaw, the light beam was turned off immediately. The duration between the start of the noxious heating and the paw withdrawal reaction was recorded as paw withdrawal latency (PWL). A pain response was interpreted as positive if the rat exhibited abrupt paw withdrawal, shaking and licking. The machine would be cut off after 40 seconds to avoid paw tissue damage by heat. Three repetitive measurements were taken at an interval of five minutes. The average PWL of the three repetitive examinations was calculated for each rat on days 0 (pre-injury) and 1, 3, 5, and 7 post-injury. The data of day 0 (pre-injury) served as the baseline. All the thermal hyperalgesia tests were conducted with two investigators who were unwitting of the grouping and treatment.

### 2.6 Measurement of Antioxidant Enzymes

The freshly removed dorsal spinal cord tissues were disrupted by homogenization in 9 volumes of cold normal saline (4°C). After centrifugation at 12,000 rpm for 15 min at 4°C, the supernatants were harvested and stored at -80°C for later analysis of CAT, SOD, GPx and total antioxidant capacity (TAC) measurements. The protein concentration of the supernatants was measured using the BCA protein assay kit (Thermo Fisher Scientific, USA). All the biochemical assays mentioned above were carried out with the relevant assay kits (Abbkine Inc., China).

### 2.7 Tissue Preparation and Immunohistochemistry

Tissue preparation and immunohistochemistry were operated as described in our previous study [40]. Briefly, the C6-8 segments of the spinal cord and C6 DRGs were harvested and then post-fixed for 24 h (spinal cord segments) and 2 h (DRGs), respectively, dehydrated for 2-3 days. The C6-8 spinal cord transverse sections (25 μm) and DRGs (10 μm) were dissected by cryostat (Leica CM1850, Leica Microsystems GmbH, Wetzlar, Germany). After blocking with 5% bovine serum albumin (BSA) at room temperature (RT) for 1 h, the spinal cord tissue slices and sections of DRGs were incubated with different primary antibodies: rabbit anti-ionized calcium binding adaptor molecule 1 (Iba1; 1:1000, Wako), mouse anti-glial fibrillary acidic protein (GFAP; 1:1000, Sigma), mouse anti-CD68 (ED-1, 1:1000, Bio-Rad), rabbit anti‐P2X3 (P2X3, 1:1000, Sigma), rabbit anti‐P2X4 (P2X4, 1:200, Alomone Labs), and rat anti-glial fibrillary acidic protein (GFAP; 1:1000, Sigma) for overnight at RT. Then, the slices were incubated with the corresponding secondary antibodies conjugated with Alexa Fluor-488 or 594 (1:1000, Invitrogen) for 4 h at RT in the dark. Finally, fluorescent images were obtained using a Zeiss fluorescence microscope (Zeiss, Gottingen, Germany) equipped with an ORCA-Flash 4.0 v2 digital CMOS camera (Hamamatsu Photonics, Iwata City, Japan).

### 2.8 Cell Culture and Drug Treatment

BV-2 cells (murine microglial cell line) were cultured in Dulbecco’s modified Eagle medium (DMEM, Gibco, USA) supplemented with 10% fetal bovine serum (FBS, Gibco, Brazil) and 1% penicillin-streptomycin (PS) at 37°C in a 5% CO_2_ incubator. BV-2 cells were plated in a 24-well microplate at a density of 1.0×10^4^ cells/well at 37°C. The cells were incubated at 37°C for 24 h, then treated with different concentrations of QCN (10, 20 and 40 μM) or Go6976 (1 μM, PKC inhibitor) for 1 h. H_2_O_2_ at a final concentration of 400 μM was then added into the medium for an additional 23 h.

### 2.9 Cell Viability Assay

To evaluate the effects of QCN on the viability of BV-2 microglia, the BV-2 cells were initially cultured at 37°C for 24 h at a density of 5.0×10^3^ cells/well and thereafter incubated with different concentrations of QCN (0, 10, 20, 40, 60 and 80 μM) for 24 h. At the end of drug treatment, the cell viability was determined using a CCK-8 assay kit according to the manufacturer’s instructions. In this assay, each well of the BV2 cells was incubated with 10 μL CCK-8 reagent for 2 h. An automatic fluorescence microplate reader was used to measure the absorbance at 450 nm wavelength to acquire the optical density (OD) value for each well. The outcomes were calculated by the following equation:

Cell viability = (OD_treatment_ - OD_blank_) / (OD_control_ - OD_blank_)

### 2.10 Intracellular ROS Measurement

Intracellular ROS was estimated using H2DCF-DA (Thermo Fisher Scientific, USA) according to previously reported methods [43, 44]. Briefly, BV-2 microglial cells were plated at 5.0×10^3^ cells/well in 96-well plates overnight. The cells were incubated with different concentrations of QCN (10, 20 and 40 μM) for 1h and subsequently stimulated with H_2_O_2_ (200 μM) for an additional 23h, then washed with PBS, and the media were replaced with 5 mM H2DCF-DA in DMEM. After that, the cells were cultured at 37°C for 30 min and then washed with PBS three times. The fluorescence intensity from the cells of each well was measured using 485 nm excitation and 535 nm emission by a FLUOstar OPTIMA microplate reader (BMG Labtech, Offenburg, Germany).

### 2.11 Western Blotting Analysis

The C7 dorsal spinal cord tissues OR BV-2 cells were processed as described in our previous study [40]. Briefly, after centrifugation at 12,000 rpm at 4^o^C for 15 min, the supernatants were collected. Protein concentrations were determined using the BCA protein assay kit (Thermo Fisher Scientific, USA). The protein lysates were separated using 10% SDS-PAGE, then electrophoretically transferred onto the PVDF membranes (Roche Applied Science, Germany). After blocking with 5% nonfat milk in TBS-T for 1 h at RT, membranes were incubated with the specific antibodies: mouse anti-protein kinase C (PKC, 1:500, Santa Cruz), rabbit anti-phospho-extracellular signal-regulated kinase (p-ERK, 1:1000, Cell Signaling Technology), rabbit anti-ERK (1:1000, Cell Signaling Technology), mouse anti-phospho-c-jun (p-c-jun, 1:500, Santa Cruz), mouse anti-c-jun (1:500, Santa Cruz), mouse anti-phospho-c-jun N-terminal kinase (p-JNK, 1:500, Santa Cruz), mouse anti-JNK (1:500, Santa Cruz), mouse anti-gp91phox (1:500, Santa Cruz), mouse anti-p22phox (1:5000, Santa Cruz), rabbit anti-p47phox (1:500, ABclonal), mouse anti-p67phox (1:500, Santa Cruz) and rabbit anti-GAPDH (1:10000, Abcam) for overnight at 4°C, and then incubated with the secondary antibodies for 1 h at room temperature. The protein bands were quantified by Image J software, using GAPDH as the internal control.

### 2.12 Statistical Analysis

Data were expressed as the mean ± standard error of the mean (SEM). Group differences in the mechanical allodynia test and thermal hyperalgesia test were analyzed using two-way analysis of variance (ANOVA) with repeated measures, with the factors being post-operative day and drug treatment. Group differences in other experiments were analyzed using one-way ANOVA followed by Dunnett’s test using SPSS 26.0 software (IBM, New York, USA). The value *p* < 0.05 was considered statistically significant.

## 3 RESULTS

### 3.1 Effects of QCN on the Body Weight and Mechanical Allodynia of BPA Rats

As shown in Fig. (**1A**), no significant differences in the body weight were observed in all surgery groups when compared to the sham control group (*F*(4, 200) = 0.6782, *p* > 0.05), suggesting that QCN exhibited no overt side effect during the 7 experimental days.

For allodynia, the withdrawal threshold in response to a rigid probe was used to measure the sensitivity to a non-harmful stimulus after injury. A significant difference was observed in the withdrawal threshold between post-operative day (*F* (3, 25) = 244.6, *p* < 0.01) and between treatment (*F* (4, 25) = 364.7, *p* < 0.001), with significant interaction observed between post-operative day and treatment (*F* (16, 100) = 43.89, *p* ≤ 0.001). As shown in Fig. (**1B**), there was no significant difference between all five groups in the value of the basal tactile pain domain before avulsion (*p* > 0.05). The withdrawal thresholds maintained unchanged in the sham operation group over the whole experimental period. Compared with the sham-operated control group, the vehicle group showed a significant decrease in mechanical pain thresholds from days 1-7 (*F* (4, 25) = 364.7, *p* < 0.001 for day 1; *F* (4, 25) = 164.74, *p* < 0.001 for day 3; *F* (4, 25) = 256.24, *p* < 0.001 for day 5; *F* (4, 25) = 237.97, *p* < 0.001 for day 7), indicating that neuropathic pain was induced in the vehicle control group. Upon immediate administration with QCN (25, 50 and 100 mg/kg) after surgery, the mechanical allodynia values were increased significantly (*p* < 0.001 for all), as compared with the vehicle control group.

As for hyperalgesia, the withdrawal latency was observed to evaluate sensitivity to a noxious heat stimulus after BPA injury. A significant difference was noted in the withdrawal threshold between post-operative day (*F* (3, 25) = 33.4, *p* < 0.001) and between treatment (*F* (4, 25) = 3.32, *p* < 0.05), with significant interaction observed between post-operative day and treatment (*F* (16, 100) = 2.61, *p* < 0.01). As depicted in Fig. (**1C**), there was no significant difference between all the five groups in the value of the basal withdrawal latencies to a thermal stimulus before avulsion (*F* (4, 25) =1.38, *p* > 0.05). After surgery, the spinal root avulsion group displayed no significant difference in ipsilateral withdrawal latencies, when compared to operated groups at any time point (*F* (4, 25) = 4.46, *p* > 0.05 for day 1; *F* (4, 25) = 6.86, *p* > 0.05 for day 3; *F* (4, 25) = 0.60, *p* > 0.05 for day 5; *F* (4, 25) = 0.44, *p* > 0.05 for day 7), although there was a trend that the withdrawal latencies dropped after surgery, especially at day 1and 3, suggesting thermal hypersensitivity was absent in 1 week following C7 spinal root avulsion.

### 3.2 Effects of QCN on the Antioxidant Enzyme Expressions of BPA Rats

The expressions of antioxidant enzymes, such as CAT, GPx and SOD, are commonly used as parameters to assess the antioxidant abilities of organisms [45, 46]. As depicted in Figs. (**2A-D**), in comparison with the sham group, the expressions of CAT, GPx, SOD and TAC in the C7 dorsal spinal cord of the vehicle group significantly decreased (*p <* 0.001 for all), indicating that the oxidative stress could be one of the mechanisms causing neuropathic pain. However, the decreased activities of CAT, GPx and SOD were efficaciously reversed by the administration of QCN (25, 50 and 100 mg/kg), when compared with the vehicle group (*F* (4, 35) = 180.48, *p* < 0.001 for CAT; *F* (4, 35) = 321.22, *p* < 0.001 for GPx; *F* (4, 35) = 82.42, *p* < 0.001 for SOD). Treatment with QCN (25, 50 and 100 mg/kg) also markedly increased the activity of TAC in the C7 dorsal spinal cord of BPA rats (*F* (4, 35) = 60.02, *p* < 0.01, *p* < 0.001 and *p* < 0.001, respectively), as compared with the vehicle group. These results unequivocally demonstrated that QCN could reduce oxidative stress in the spinal cord lesion site by enhancing the activities of antioxidant enzymes.

### 3.3 Suppressive Effects of QCN on Macrophages in DRG of BPA Rats

We stained C6 DRG using Iba-1 to detect whether there was any change of macrophages in quantity after the avulsion of the spinal nerve root. In response to BPA injury, macrophages were activated. Figs. (**3A** and **B**) showed that marked differences in the number of macrophages were noted in the C6 DRG of the vehicle group, when compared with that in the sham group (*F*(4, 25) = 227.38, *p <* 0.001). When compared to the vehicle control group, oral administration of QCN (25, 50 and 100 mg/kg) for 7 days significantly reduced the macrophage activation (*p* < 0.01, *p* < 0.001 and *p* < 0.001, respectively).

### 3.4 Effects of QCN on Neuroinflammatory Response in the Spinal Dorsal Horn of BPA Rats

**Fig. (4):**
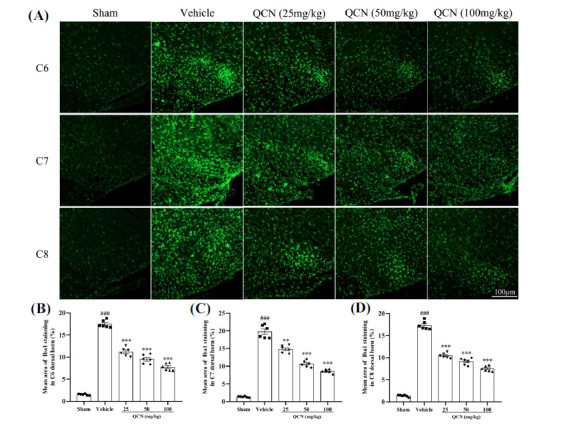
Effects of QCN on the microglia activity in the spinal dorsal horn of BPA rats. (**A**) Representative images of Iba1 staining cells in the ipsilateral C6-8 dorsal spinal segments at day 7 post-avulsion injury. Mean Iba1-immunoreactive area in the ipsilateral (**B**) C6, (**C**) C7 and (**D**) C8 dorsal spinal segments from all groups. Data were expressed as the mean ± SEM (n = 6). ^###^*p <* 0.01 *vs*. the sham control group; **p < 0.01 and ***p < 0.001 *vs*. the vehicle control group. Scale bar: 100 μm.

**Fig. (5):**
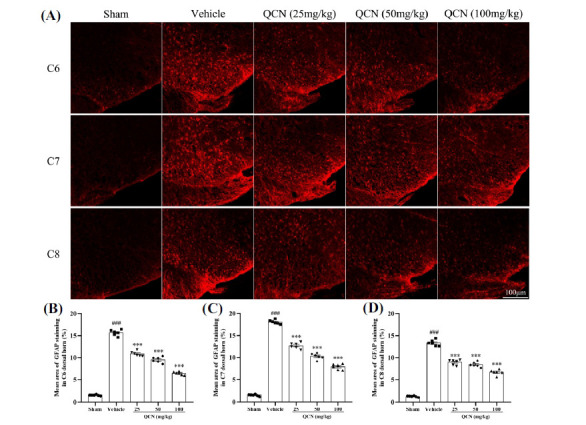
Effects of QCN on the astrocyte activity in the spinal dorsal horn of BPA rats. (**A**) Representative images of GRAP staining cells in the ipsilateral C6-8 dorsal spinal segments at day 7 post-avulsion injury. Mean GFAP-immunoreactive area in the ipsilateral (**B**) C6, (**C**) C7 and (**D**) C8 dorsal spinal segments from all groups. Data were expressed as mean ± SEM (n = 6). ^###^*p <* 0.001 *vs*. the sham control group; ***p < 0.001 *vs*. the vehicle control group. Scale bar: 100 μm.

**Fig. (6):**
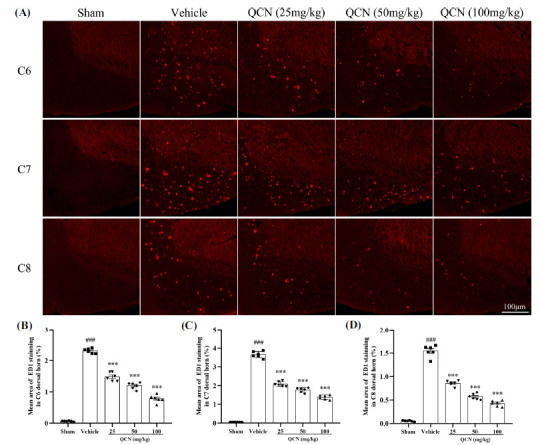
Effects of QCN on the macrophage activity in the spinal dorsal horn. (**A**) Representative images of ED1 staining cells in the ipsilateral C6-8 dorsal spinal segments at day 7 post-avulsion injury. Mean ED1-immunoreactive area in the ipsilateral (**B**) C6, (**C**) C7 and (**D**) C8 dorsal spinal segments from all groups. Data were expressed as mean ± SEM (n = 6). ^###^*p <* 0.001 *vs*. the sham control group; ***p < 0.001 *vs*. the vehicle control group. Scale bar: 100 μm.

At day 7 after avulsion, we observed dramatic microglia (Iba1), astrocytes (GFAP) and macrophages (ED1) expressions in the ipsilateral C6 to C8 dorsal horns of the spinal cord (particularly in C6 and C7 segments) in the vehicle control group, when compared to the sham control group (Figs. **4-6** (**A-D**)). Nevertheless, in comparison with the vehicle-treated control group, Iba1, GFAP and ED1 (F(4, 25) = 297.49, *p <* 0.001 for Iba1; F(4, 25) = 250.85, *p <* 0.001 for GFAP; F(4, 25) = 445.59, *p <* 0.001 for ED1 in C6 spinal dorsal horns); ((F(4, 25) = 614.64, *p <* 0.001 for Iba1; F(4, 25) = 717.69, *p <* 0.001 for GFAP; F(4, 25) = 377.16, *p <* 0.001 for ED1 in C7 spinal dorsal horns); (F(4, 25) = 423.31, *p <* 0.001 for Iba1; F(4, 25) = 660.64, *p <* 0.001 for GFAP; F(4, 25) = 358.40, *p <* 0.001 for ED1 in C8 spinal dorsal horns) staining in the C6-8 spinal dorsal horns were significantly decreased in the QCN-treated rats (25, 50 and 100 mg/kg), especially in the C7 dorsal horn (*p <* 0.01 for all). These data revealed that QCN administration exerted a potent suppressive effect on the neuroinflammatory reactivity by attenuating the activation of microglia, astrocytes and macrophages at the spinal dorsal horn, thereby resulting in the amelioration of the BPA pain injury.

### 3.5 Effects of QCN on P2X3 and P2X4 Expressions in DRG of BPA Rats

**Fig. (7):**
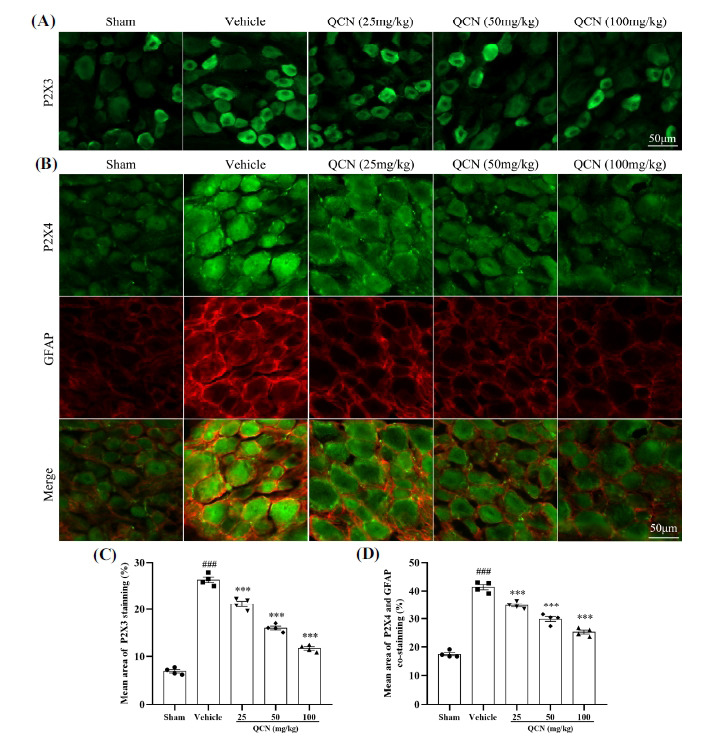
Effects of QCN on P2X3 and P2X4 levels in DRG. Representative images of (**A**) P2X3 staining, (**B**) P2X4 (green) and GFAP (red) co-staining in the ipsilateral C6 DRG at day 7 post-avulsion injury. Mean (**C**) P2X3-immunoreactive area, (**D**) P2X4 and GFAP co-immunoreactive area in the ipsilateral C6 DRG from all groups. Data were expressed as mean ± SEM (n = 4). ^###^*p <* 0.001 *vs*. the sham control group; ***p < 0.001 *vs*. the vehicle control group. Scale bar: 50 μm.

Immunofluorescence was used to evaluate the P2X3 production in DRG at day 7 after BPA injury (Figs. **7A** and **C**). This analysis revealed that in comparison to the sham control animals, rats in the vehicle group had significantly higher P2X3 generation in C6 DRG at day 7 (*F*(4, 15) = 254.28, *p <* 0.001). However, as compared to the vehicle control group, continual gastric infusion with QCN (25, 50 and 100 mg/kg) for 7 days obviously reduced the P2X3 expression in DRG of BPA rats (*p <* 0.001 for all).

Besides, the co-expression of P2X4 receptor and GFAP in the C6 DRG of BPA rats were tested using double immunofluorescence (Figs. **7B** and **D**). Upregulation of GFAP is regarded as a marker of satellite glial cell (SGC) activation in DRG [38, 47]. Double-labeling immunostaining results indicated that P2X4 was co-expressed with GFAP in the C6 DRG. The co-expression levels of P2X4 and GFAP in vehicle control group rats were boosted (*F*(4, 15) = 143.05, *p <* 0.001), as compared with the sham group. Nonetheless, the P2X4 and GFAP co-expression levels in the BPA rats treated with QCN group (25, 50 and 100 mg/kg) for 7 days were markedly downregulated relative to those in the vehicle-treated BPA group (*p <* 0.001 for all). These results indicated that QCN decreased the SGC activation and lowered the expression of the P2X4 receptor.

### 3.6 Effects of QCN on PKC/MAPK Pathway in the Spinal Cords of BPA Rats

**Fig. (8):**
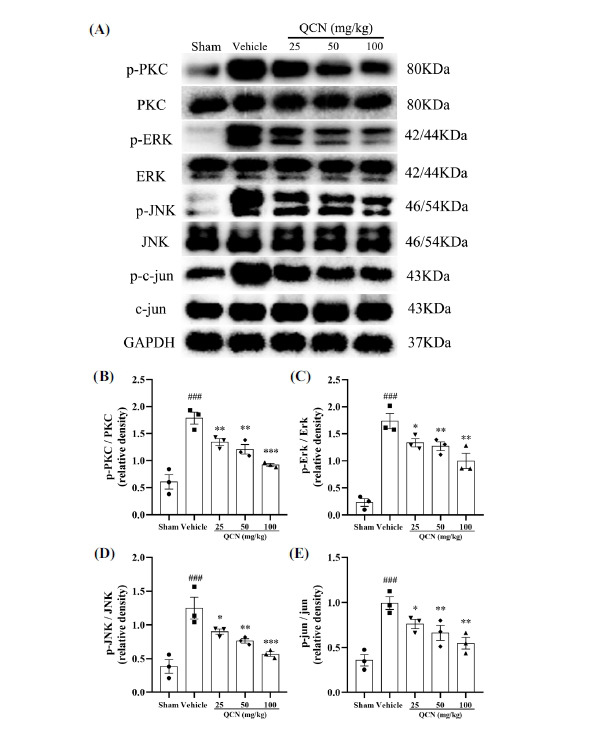
Effects of QCN on PKC/MAPK signaling pathway in the spinal cords of BPA rats. (**A**) The protein levels of p-PKC, p-ERK, p-JNK and p-c-jun were determined by Western blot. Quantification of (**B**) p-PKC to PKC, (**C**) p-ERK to ERK, (**D**) p-JNK to JNK and (**E**) p-c-jun to c-jun proteins in the dorsal horn of the C7 spinal segments. Data were expressed as mean ± SEM (n = 3). ^###^*p <* 0.001 *vs*. the sham control group; *p < 0.05, **p < 0.01 and ***p < 0.001 *vs*. the vehicle control group.

Among the major upstream regulators of ERK activity, protein kinase C (PKC) is a family of enzymes deeply involved in pain transmission [48]. Our western blot results showed that, on day 7 after BPA surgery, the ratios of protein expression including p-PKC/PKC (*F*(4, 10) = 23.26, *p* < 0.001), p-ERK/ERK (*F*(4, 10) = 27.62, *p* < 0.001), p-JNK/JNK (*F*(4, 10) = 13.55, *p* < 0.001) and p-c-jun/c-jun (*F*(4, 10) = 12.27, *p* < 0.001) were clearly accentuated in the vehicle group, as compared with the sham control group. Treatment with QCN (25, 50 and 100 mg/kg) could explicitly decrease the relative levels of p-PKC/PKC (*p* < 0.01, *p* < 0.01 and *p* < 0.001, respectively), p-ERK/ERK (*p* < 0.05, *p* < 0.01 and *p* < 0.01, respectively), p-JNK/JNK (*p* < 0.05, *p* < 0.01 and *p* < 0.001, respectively) and p-c-jun/c-jun (*p* < 0.05, *p* < 0.01 and *p* < 0.01, respectively) in the spinal dorsal horns of BPA rats, as compared with the vehicle control group (Fig. **8A-E**). All these results unambiguously indicated that QCN was capable of regulating the protein expressions of PKC and the related downstream molecules in the dorsal spinal cords of BPA rats, sequentially alleviating the neuropathic pain after avulsion injury.

### 3.7 Effects of QCN on NADPH Oxidase Enzymes in the Spinal Cords of BPA Rats

Previous studies have suggested that NADPH oxidase enzymes (NOX) are important ROS sources during pain sensitization, and excessive ROS production gives rise to severe neuropathic pain in avulsion injury [49, 50]. In this experiment, we determined whether QCN administration could suppress the oxidative damage in the spinal cord following avulsion-induced pain. As shown in Figs. (**9A-E**), at day 7 after avulsion, the protein expressions of NOX subunits, including gp91phox (*F*(4, 10) = 11.61, *p* < 0.01), p22phox (*F*(4, 10) = 10.41, *p* < 0.01), p47phox (*F*(4, 10) = 33.33, *p* < 0.001), and p67phox (*F*(4, 10) = 12.31, *p* < 0.01), were significantly ascendant in the vehicle control group, when compared with the sham control group. At the same time, QCN (50 and 100 mg/kg) significantly decreased the relative protein levels of gp91phox (*p* < 0.05 and *p* < 0.01, respectively), p22phox (*p* < 0.05 and *p* < 0.01, respectively), p47phox (*p* < 0.01 and *p* < 0.001, respectively) and p67phox (*p* < 0.01 and *p* < 0.01, respectively) in the spinal dorsal horns of BPA rats, as compared with the vehicle control group. All these results unambiguously indicated that QCN was potent in inhibiting the protein expressions of NADPH pathway-related oxidases in the dorsal spinal cords of BPA rats, thus conferring analgesic efficacy on avulsion injury through mitigating oxidative stress.

### 3.8 Effects of QCN on the Cytotoxicity and ROS Production Induced by H_2_O_2_ in BV-2 Cells

In our experiment, CCK8 assay was performed to determine the potential cytotoxicity of QCN in BV-2 cells. As depicted in Fig. (**10A**), after treatment with QCN at the doses of 10, 20 and 40 μM for 24 h, no reduction in the cell viability was found, as compared with the control group, demonstrating that QCN was not cytotoxic to BV-2 cells at concentrations up to 40 μM. In order to ascertain the protective functions of QCN in the H_2_O_2_-stimulated BV-2 cells, cells were then pretreated with QCN (10, 20 and 40 μM) for 1 h followed by treatment with H_2_O_2_ (400 μM) for a further 23 h. As demonstrated in Fig. (**10B**), H_2_O_2_ (400 μM) treatment significantly reduced the cell viability in BV-2 cells (*F*(4, 25) = 203.69, *p* < 0.01), as compared with the control group. However, pretreatment with QCN (10, 20 and 40 μM), especially at the concentration of 20 and 40 μM, significantly weakened the cell death induced by H_2_O_2_ in BV-2 cells (*p* < 0.01 for all), as compared to those not treated with QCN.

The underlying anti-oxidative capacity of QCN was observed against the production of ROS in the H_2_O_2_-stimulated BV-2 cells. On the strength of the above results, the QCN at concentrations of 10, 20 and 40 μM were chosen to estimate the intracellular formation of ROS using H2DCF-DA. As shown in Fig. (**10C**), H_2_O_2_ alone markedly augmented the ROS production (F(4, 25) = 355.79, *p* < 0.01), as compared to the control group. Nevertheless, pretreatment with QCN (10, 20 and 40 μM), especially at the concentration of 20 and 40 μM, significantly reduced the level of ROS (*p* < 0.01 for all) in the H_2_O_2_-stimulated BV-2 cells, as compared with the H_2_O_2_-treated control group. Based on these results, the effects of QCN (20 and 40 μM) on the protein expression of NOX were determined by western blot analysis.

### 3.9 Effects of QCN on the H_2_O_2_-induced Activation of PKC/MAPK/NOX Pathway in BV-2 Cells

As shown in Figs. (**11A-G**), the protein expression of the ratios of p-PKC/PKC (*F*(4, 10) = 10.29, *p* < 0.001) and p-ERK/ERK (*F*(4, 10) = 6.73, *p* < 0.01), as well as NOX subunits, including gp91phox (*F*(4, 10) = 7.15, *p* < 0.001), p22phox (*F*(4, 10) = 7.61, *p* < 0.01), p47phox (F(4, 10) = 8.18, *p* < 0.001), and p67phox (*F*(4, 10) = 8.12, *p* < 0.001) were significantly increased in the H_2_O_2_-stimulated BV-2 cells, as compared with the control group. However, pretreatment with QCN (20 and 40 μM) significantly reduced the protein levels of p-PKC/PKC (*p* < 0.05 and *p* < 0.01, respectively), p-ERK/ERK (*p* < 0.05 for both), gp91phox (*p* < 0.05 for both), p22phox (*p* < 0.05 and *p* < 0.01, respectively), p47phox (*p* < 0.05 and *p* < 0.01, respectively), and p67phox (*p* < 0.05 for both) in the H_2_O_2_-treated BV-2 cells, as compared with the vehicle control group. Pretreatment with Go6976 (1 μM, PKC inhibitor) also inhibited the ratios of p-PKC/PKC (*p* < 0.01) and p-ERK/ERK (*p* < 0.01), as well as gp91phox (*p* < 0.01), p22phox (*p* < 0.01), p47phox (*p* < 0.01), and p67phox (*p* < 0.01) in the H_2_O_2_-induced BV-2 cells, as compared with the vehicle control group. Interestingly, QCN at the concentration of 40 μM exerted a similar inhibitory effect to that of Go6976 (PKC inhibitor) on these proteins. These results manifest that the protective effects of QCN on H_2_O_2_-stimulated BV-2 cells are PKC/MAPK/NOX pathway dependent.

## 4 DISCUSSION

Neuropathic pain as a consequence of BPA constitutes an important additional burden on the life quality of patients who are already suffering from sensory, motor, and autonomic defects [20]. Neuropathic pain treatment becomes a central challenge owing to the fact that this type of pain is notoriously unresponsive to most available medications [7, 51]. QCN has been confirmed to possess a spectrum of advantageous effects, especially its higher antioxidant capacity relative to many other flavonoids. The cardinal feature regarding the chemical structure of QCN is the several OH groups in its structure which can bond to ROS and conserve cells viability [52, 53].

Our laboratory has established a credible animal model to study neuropathic pain through the avulsion of the C7 spinal root by intravertebral pattern [54]. Other animal models, covering chronic constriction injury, spared nerve injury, spinal nerve ligation, partial sciatic nerve ligation, and rhizotomy models have also been applied for experimental explorations on neuropathic pain [55-58]. However, these models merely imitate the clinical symptoms of neuropathic pain occurred in humans, but fall short of mimicking the underlying pathogenesis of neuropathic pain. In comparison with other animal models of peripheral neuropathy, BPA rat model presents some explicit advantages. Firstly, the autotomy, which is often reported in the most of other neuropathic pain animal models [59], was not found in the avulsion of the C7 brachial plexus. Secondly and also the most crucial advantage, the major characteristics of BPA are the speedy initiation of pain (a response which takes place right away after the trauma) and the enduring neuropathic development, which can be detected even remotely from the lesion site. Thirdly, this avulsion model does not lead to thermal hyperalgesia, which is generally detected in other animal models [60, 61]. Therefore, our present study aimed to determine the protective effects of QCN on neuropathic pain using the C7 spinal root avulsion-induced neuropathic pain in adult rats.

Previous studies have certified that the neuropathic pain associated with the BPA model in rats elicits long-range mechanical allodynia [62]. Allodynia is an arresting feature in patients with neuropathic pain [63]. We attested for the first time in this study that immediate administration of QCN for 7 days remitted the onset of pain behavior associated with mechanical hyperalgesia in the right front paws after C7 BPA injury. The transmission of pain messages is mediated by multiple changes in both the PNS and CNS [64-66]. Nerve injury elicits activation of intrinsic immune cells (*e.g*. macrophages), aggregation of glial cells (*e.g*. microglia and astrocyte) to the damage site, and discharge of inflammatory mediators, such as cytokines, chemokines, ATP and ROS, thus triggering peripheral and central sensitization [67, 68]. Prominently, activated microglia are known to result in the activation of astrocytes [69], which in turn liberates signaling molecules that play an impact on microglia. This astrocyte-microglia crosstalk is a momentous specialty in the pathophysiology of neuropathic pain [70].

In our present study, the rats treated with QCN showed a notable decrease in activated macrophages, microglia cells and astrocytes dose-dependently at day 7 after nerve root avulsion injury. The consequences showed that the mitigation effects of QCN against pain induced by BPA injury are tightly related to its anti-inflammatory effect. In addition, ATP liberating from injured cells in peripheral tissues intervenes in the activation of P2X receptors on the cell surface, subsequently bringing about inflammation [71, 72]. P2X receptor antagonists are proverbial to refrain mechanical allodynia in both rats and mice [73, 74]. Neuropathic pain is generally a result of somatosensory nervous system damage, leading to the release of colossal amounts of ATP. Consequently, the expression of P2X3 and P2X4 is overtly increased in DRGs, as evidenced by high levels of P2X3 and incremental co-expression of P2X4 and GFAP in this finding, and their suppressions relieve mechanical hyperalgesia in neuropathic pain animal models [75-80]. In our experiments, QCN treatment dose-dependently and dramatically ameliorated the P2X3 level in DRG as well as the P2X4 and GFAP co-expression in the DRG SGCs in neuropathic pain rats elicited by avulsion, thereby blocking the excessive secretion of ATP and attenuating the inflammatory response.

Oxidative stress is believed to play a pivotal role in the development and continuity of neuropathic pain [81, 82]. Excessive ROS (hydrogen peroxide, superoxide, and the active superoxide byproduct) has a detrimental effect on organelles, antioxidant defenses and other biomolecules, bringing about mitochondrial dysfunction, glial activation and inflammatory reaction. This detrimental environment is eventually responsible for the representative algetic symptoms of neuropathic pain [81]. In the spinal dorsal horn of neuropathic pain animals, ROS has been shown to contribute to the enhancement of pain behavior [83]. The significant decrease in antioxidant enzymes, including CAT, GPx, SOD, and TAC after BPA in this study implies that oxidative stress is one of the mechanisms of neuropathic pain. As mentioned above, QCN has the immense antioxidant capacity and is a powerful ROS scavenger, and our present study also confirmed its antioxidant effect. We found that the reduced activities of CAT, GPx, SOD and TAC by BPA were rapidly upturned by QCN administration in a dose-dependent manner. ROS are generated intracellularly through multifarious mechanisms, one of which is NADPH oxidase enzymes (NOX) [84]. NOX is expressed chiefly in phagocytic cells. However, all the major cell categories in the brain (neurons, astrocytes and microglia) constitutively secrete multiple NOX isoforms, including gp91phox, p22phox, p47phox, and p67phox and its associated proteins, which mediate neuropathic pain processing [50, 85, 86]. Some cellular signals have been considered to modulate the NADPH oxidase activity, such as MAPKs (ERK1/2, p38 MAPK, JNK and c-jun) and PKC [86]. Further, interference withROS expression by inhibition/ablation of NADPH oxidase and elimination of ROS by applying ROS scavengers and antioxidants have been revealed to result in the inhibition of neuropathic pain [81, 87-89].

In this study, we indeed observed the increased levels of p-PKC, p-ERK, p-JNK and p-c-jun and NOX complexes gp91phox, p22phox, p47phox and p67phox in the spinal dorsal horn after the avulsion injury. However, avulsion-induced activation of these proteins was significantly inhibited by QCN administration, and these results were concordant with the reports of other investigators [89, 90]. Our experimental findings amply demonstrated that QCN was able to inhibit the activation of the PKC/MAPK/NOX pathway to suppress the oxidative damage induced by BPA in neuropathic pain rats.

Microglia, located in the CNS, play a critical role in the immune modulation of the CNS [91, 92]. After a nerve lesion, microglia in the normal status (customarily called “resting” microglia) in the spinal dorsal horn is switched to an active status through a sequence of cellular and molecular alterations. Persistent triggering of microglia by exterior signals can cause their over-activation, leading to the production of excessive amounts of ROS [93]. More specifically, several molecular mechanisms have been recognized in the H_2_O_2_-induced microglial cell-mediated oxidative damage [94, 95]. In our study, BV-2 cells (microglia cell line) [96] were treated with H_2_O_2_ (200 μM), and the concentration of H_2_O_2_ was chosen in line with the previous study [95] to evaluate the potential anti-oxidative property of QCN against the generation of ROS. The intracellular level of ROS was assessed utilizing oxidation-sensitive dye DCFH-DA as the substrate [97]. Our *in vitro* study showed that QCN significantly inhibited the ROS overproduction induced by H_2_O_2_ in BV-2 cells. In addition, PKC can trigger the activation of NADPH oxidases, which are involved in multinucleation, although *via* incremental fusion [98]. Go6976, a widely acknowledged PKC inhibitor, has been demonstrated to inhibit oxidative stress and ROS production mediated by NOX [99, 100]. Our *in vitro* experiments indicated that QCN treatment was able to inhibit the activation of PKC/NOX signaling pathway triggered by H_2_O_2_ in BV-2 cells. QCN (40 μM) showed a similar depressor effect on the relative protein expressions of PKC/NOX pathway as that of PKC inhibitor Go6976, indicating that QCN could repress oxidative damage induced by H_2_O_2_ in BV-2 cells *via* suppressing the protein expression of PKC and its downstream NOX isoforms.

## CONCLUSION

**Fig. (12):**
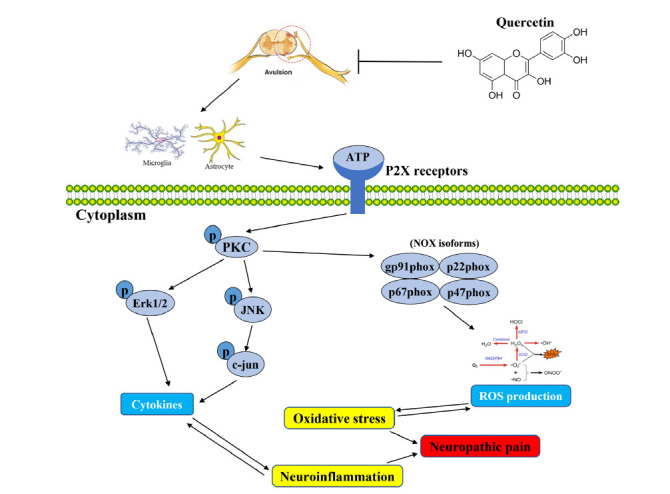
Schematic illustration depicting the putative pathways associated with the therapeutic effect of QCN on the BPA-induced neuropathic pain in rats. QCN significantly attenuate pain hypersensitivity following the C7 spinal root avulsion in rats. The neuroprotective effects of QCN are attributed to the inhibition of neuroinflammatory response and oxidative stress, at least partially *via* suppression of P2X receptors and inhibition of the activation of PKC/MAPK/NOX pathway.

In summary, our current experiments have for the first time unraveled that QCN, a well-known plant-sourced flavonoid, was able to mitigate mechanical hypersensitivity induced by BPA in neuropathic pain rats. The pain catabatic effects of QCN are attributed to the activation of the antioxidant enzymes, suppressed the activation of the macrophages, microglia and astrocytes, as well as P2X receptors, partially *via* attenuation of the activation of PKC/MAPK/NOX pathway (Fig. **12**). We believe that QCN is a promising naturally existing chemical worthy of in-depth development into a pharmacological treatment for neuropathic pain.

## Figures and Tables

**Fig. (1):**
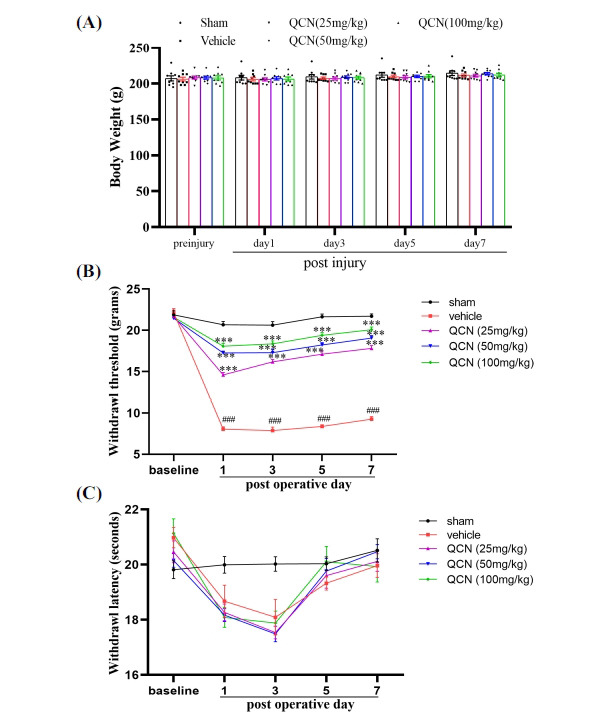
Effects of QCN on the body weight and mechanical allodynia response. (**A**) The average body weight of rats in different experimental groups (n = 9). (**B**) The changes of mechanical allodynia values (PWT) in different experimental groups (n = 6). (**C**) The changes of thermal hyperalgesia values (PWL) in different experimental groups (n = 6). Data were expressed as the mean ± SEM. ^###^*p <* 0.001 *vs*. the sham control group; ***p < 0.001 *vs*. the vehicle control group.

**Fig. (2):**
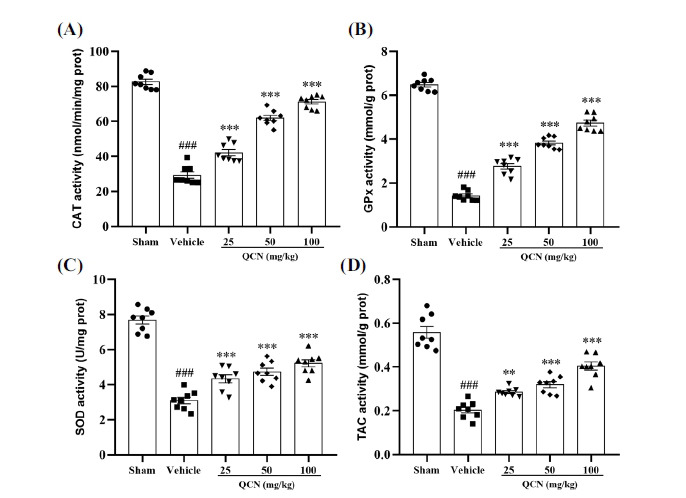
Effects of QCN on the antioxidant activities in C7 spinal dorsal horn. (**A**) CAT activity; (**B**) GPx activity; (**C**) SOD activity; (**D**) TAC activity. Data were expressed as mean ± SEM (n = 8). ^###^*p <* 0.001 *vs*. the sham control group; **p < 0.01 and ***p < 0.001 *vs*. the vehicle control group.

**Fig. (3):**
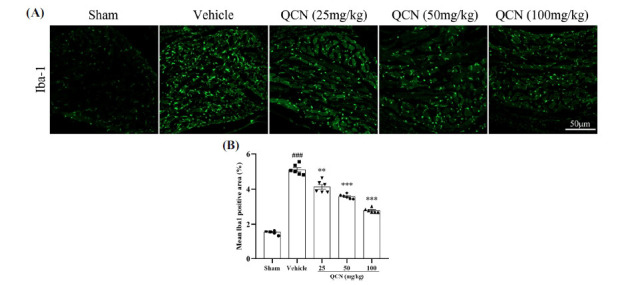
Effects of QCN on macrophages in DRG. (**A**) Representative images of Iba-1 staining in the ipsilateral C6 DRG at day 7 post-avulsion injury. (**B**) Mean Iba1-immunoreactive area in the ipsilateral C6 DRG from all groups. Data were expressed as mean ± SEM (n = 6). ^###^*p <* 0.01 *vs*. the sham control group; **p < 0.01 and ***p < 0.001 *vs*. the vehicle control group. Scale bar: 50 μm.

**Fig. (9):**
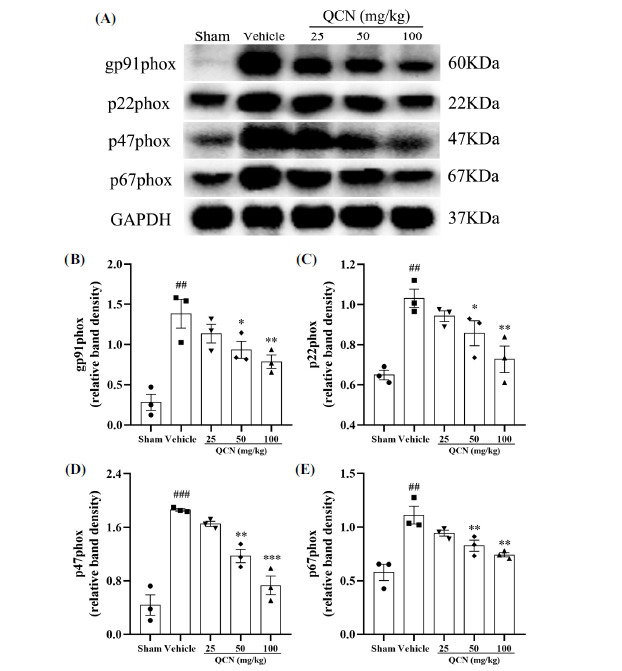
Effects of QCN on NADPH oxidase enzymes in the spinal cords of BPA rats. (**A**) The protein levels of gp91phox, p22phox, p47phox and p67phox were determined by Western blot. Quantification of (**B**) gp91phox, (**C**) p22phox, (**D**) p47phox and (**E**) p67phox proteins in the dorsal horn of the C7 spinal segments. Data were expressed as mean ± SEM (n = 3). ^##^*p <* 0.01 and ^###^*p <* 0.001 *vs*. the sham control group; *p < 0.05, **p < 0.01 and ***p < 0.001 *vs*. the vehicle control group.

**Fig. (10):**
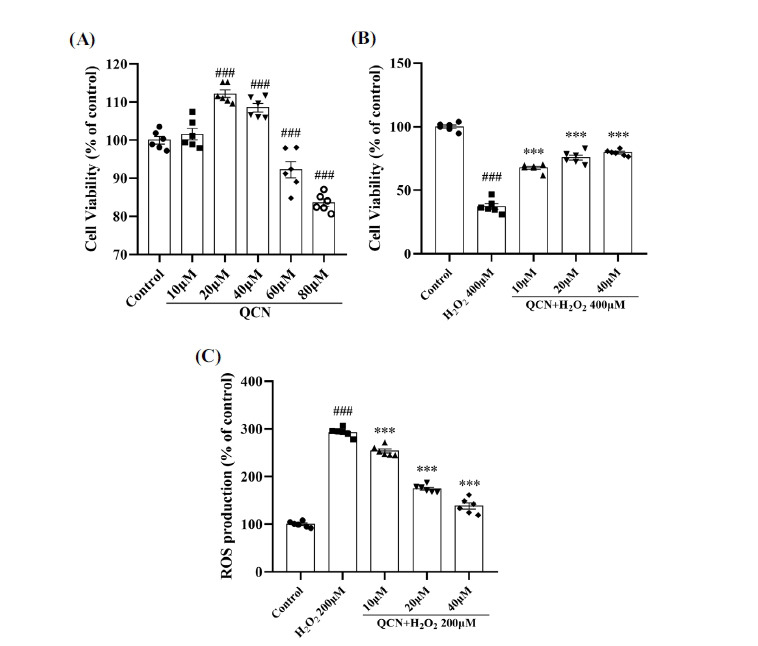
Effects of QCN on the cytotoxicity and ROS production in the H_2_O_2_-stimulated BV-2 cells. (**A**) The cell viability of BV-2 cells were measured after treatment with QCN (10, 20 30, 40, 50, 60, 70, 80 μM) for 24 h. (**B**) The cell viability of BV-2 cells were determined after pre-treatment with QCN (10, 20 and 40 μM) for 1 h followed by treatment with H_2_O_2_ (400 μM) for additional 23 h. (**C**) The ROS production of BV-2 cells pretreated with QCN (10, 20 and 40 μM) for 1 h followed by treatment with H_2_O_2_ (200 μM) for additional 23 h. Data were expressed as mean ± SEM (n = 6). ^###^*p <* 0.001 *vs*. the control group; ***p < 0.001 *vs*. the H_2_O_2_-treated control group.

**Fig. (11):**
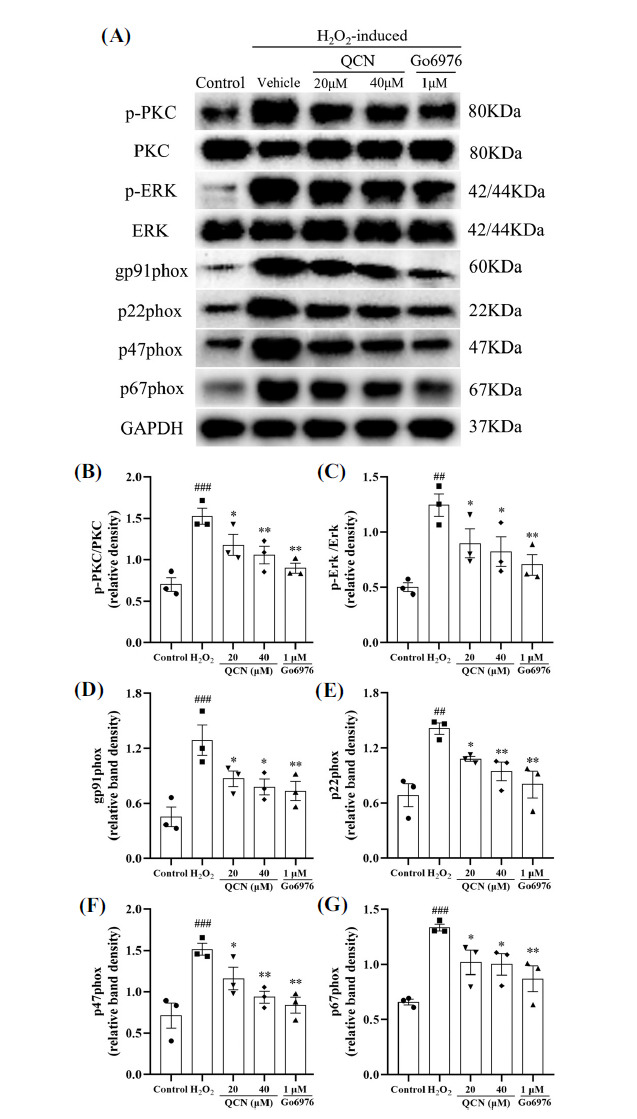
Effects of QCN on PKC/MAPK/NOX signaling pathway in the H_2_O_2_-stimulated BV-2 cells. BV-2 cells were pretreated with QCN (20 and 40 μM) or Go6976 (1 μM) for 1 h followed by treatment with H_2_O (200 μM) for additional 23 h. (**A**) The protein levels of p-PKC, p-ERK, gp91phox, p22phox, p47phox and p67phox were determined by Western blot analysis. Quantification of (**B**) p-PKC/PKC, (**C**) p-ERK/ERK, (**D**) gp91phox, (**E**) p22phox, (**F**) p47phox and (**G**) p67phox proteins in the dorsal horn of the C7 spinal segments. Data were expressed as mean ± SEM (n = 3). ^##^*p <* 0.01 and ^###^*p <* 0.001 *vs*. the control group; *p < 0.05 and **p < 0.01 *vs*. the H_2_O_2_-treated control group.

## Data Availability

The data that support the findings of this study are available from the corresponding authors upon reasonable request.

## LIST OF ABBREVIATIONS

ATP: Adenosine Triphosphate
BPA: Brachial Plexus Avulsion
CAT: Catalase
CNS: Central Nervous System
DRG: Dorsal Root Ganglion
ERK: Extracellular Signal-regulated Kinase
GFAP: Glial Fibrillary Acidic Protein
GPx: Glutathione Peroxidase
H_2_O_2_: Hydrogen Peroxide
Iba1: Ionized Calcium Binding Adaptor Molecule 1
JNK: C-jun N-terminal Kinase
MAPK: Mitogen-activated Protein Kinase
NADPH: Nicotinamide Adenine Dinucleotide Phosphate
NOX: NADPH Oxidase
P2X: Purinergic 2X Receptor
PFA: Paraformaldehyde
PKC: Protein Kinase C
PNS: Peripheral Nervous System
PWT: Paw Withdrawal Threshold
QCN: Quercetin
ROS: Reactive Oxygen Species
RT: Room Temperature
SOD: Superoxide Dismutase
TAC: Total Antioxidant Capacity
