# Supplementary material for: Quercetin Ameliorates Neuropathic Pain after Brachial Plexus Avulsion via Suppressing Oxidative Damage through Inhibition of PKC/MAPK/NOX Pathway
Source: Curr Neuropharmacol. 2023 Sep 1;21(11):2343–61. doi: 10.2174/1570159X21666230802144940 (PMC10556381; doi:10.2174/1570159X21666230802144940)
Supplement: Supplementary file 1 — Supplementary material is available on the publisher’s website along with the published article. [file CN-21-2343_SD1.pdf]

## Supplementary Material

# Quercetin Ameliorates Neuropathic Pain after Brachial Plexus Avulsion *via* Suppressing Oxidative Damage through Inhibition of PKC/MAPK/NOX Pathway

Yanfeng Huang[1], Xie Zhang[2,3], Yidan Zou[4], Qiuju Yuan[5,*], Yan-Fang Xian[1,*] and Zhi-Xiu Lin[1,6,*]

*[1]School of Chinese Medicine, Faculty of Medicine, The Chinese University of Hong Kong, Shatin, N.T., Hong Kong SAR, China; [2]Research Center for Integrative Medicine of Guangzhou University of Chinese Medicine (Key Laboratory of Chinese Medicine Pathogenesis and Therapy Research), School of Basic Medical Sciences, Guangzhou University of Chinese Medicine, Guangzhou, Guangdong. P.R. China; [3]Department of Medical Biotechnology, School of Basic Medical Sciences, Guangzhou University of Chinese Medicine, Guangzhou, Guangdong. P.R. China; [4]Department of Anaesthesia and Intensive Care and Peter Hung Pain Research Institute, Faculty of Medicine, The Chinese University of Hong Kong, Shatin, N.T., Hong Kong SAR, China; [5]Centre for Regenerative Medicine and Health, Hong Kong Institute of Science & Innovation, Chinese Academy of Sciences, Hong Kong Science Park, Shatin, N.T., Hong Kong SAR, China; [6]Hong Kong Institute of Integrative Medicine, The Chinese University of Hong Kong, Hong Kong SAR, China*

**1875-6190/XX $65.00+.00** **© XXXX Bentham Science Publishers**

## Supplementary Data

Fig. 8.

p-PKC

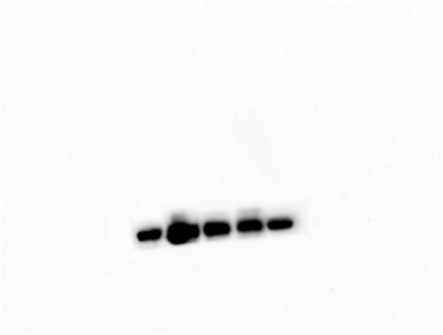

1

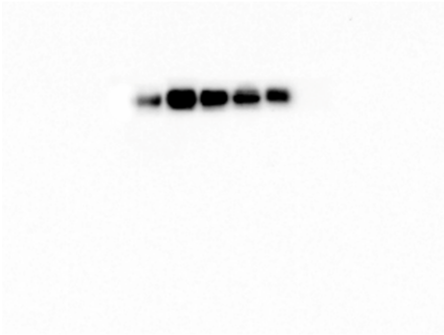

2

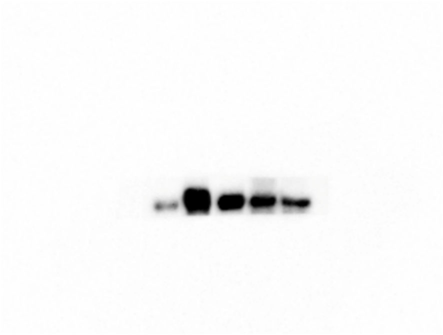

3

PKC

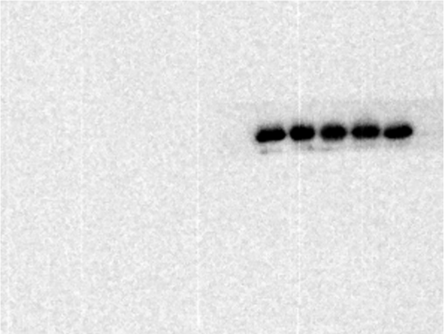

1

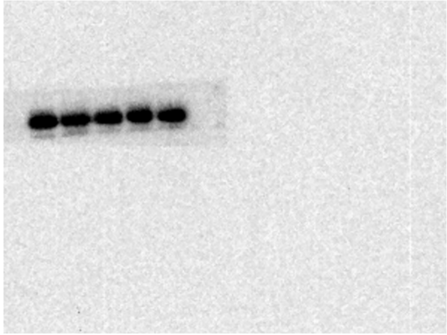

2

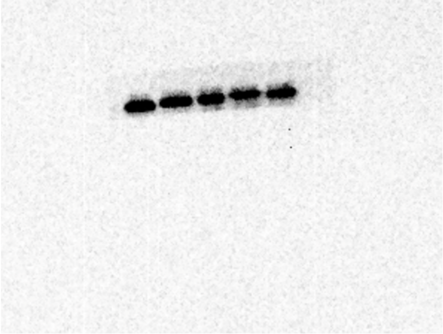

3

p-ERK

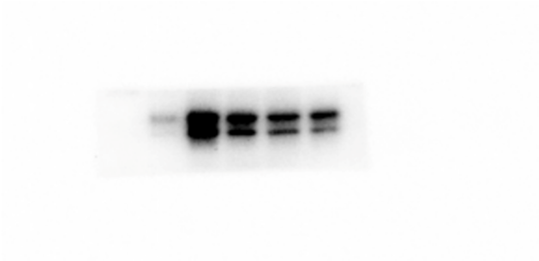

1

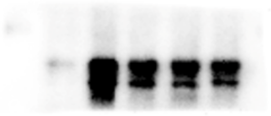

2

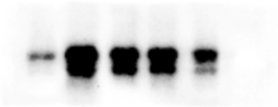

3

ERK

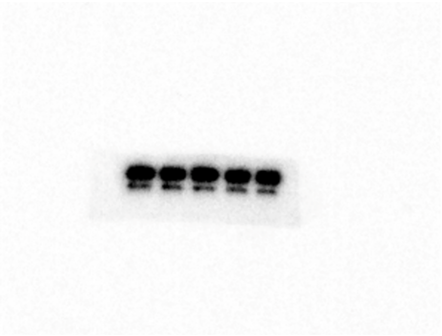

1

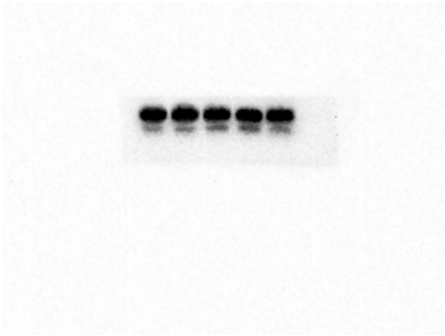

2

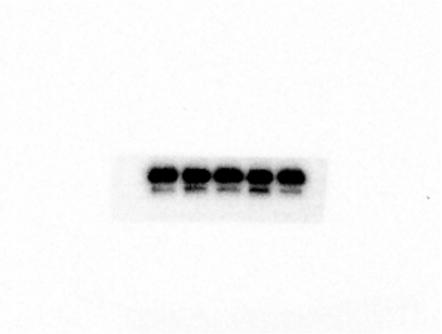

3

p-JNK

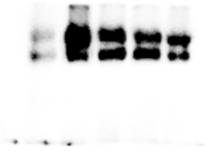

1

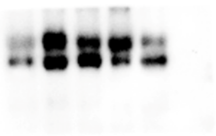

2

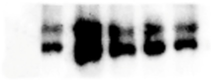

3

JNK

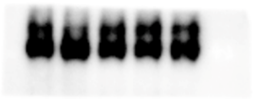

1

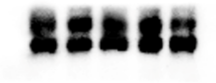

2

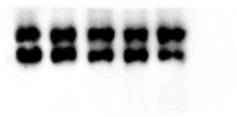

3

p-c-jun

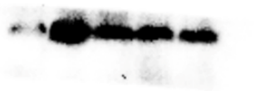

1

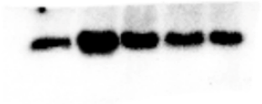

2

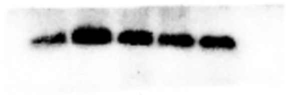

3

c-jun

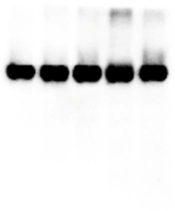

1

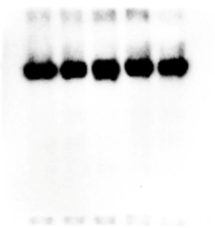

2

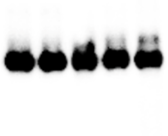

3

GAPDH

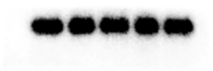

1

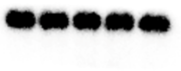

2

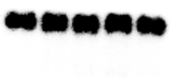

3

Fig. 9.

gp91phox

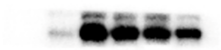

1

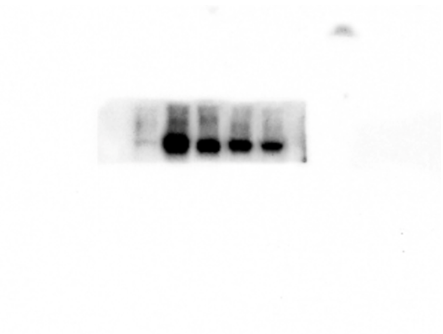

2

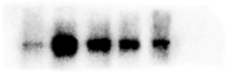

3

p22phox

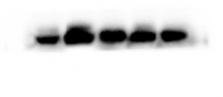

1

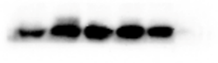

2

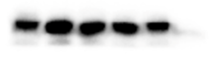

3

p47phox

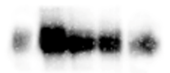

1

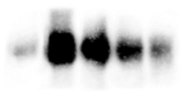

2

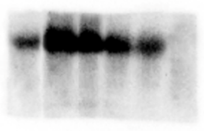

3

P67phox

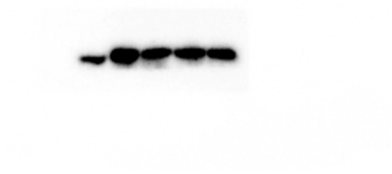

1

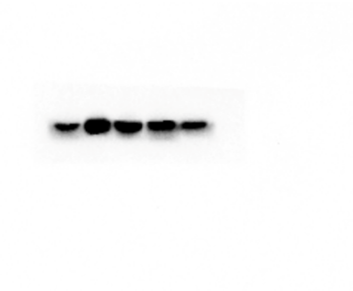

2

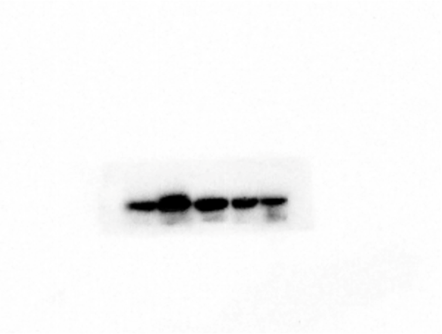

3

GAPDH

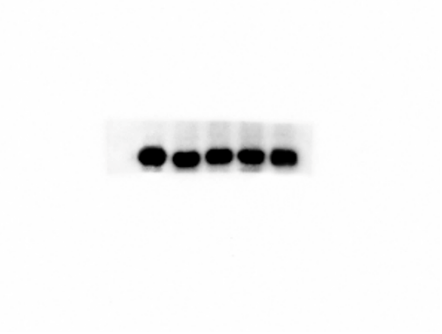

1

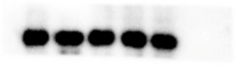

2

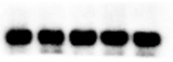

3

Fig. 11.

p-PKC

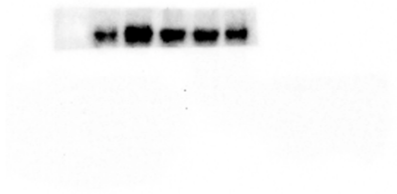

1

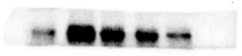

2

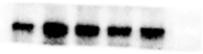

3

PKC

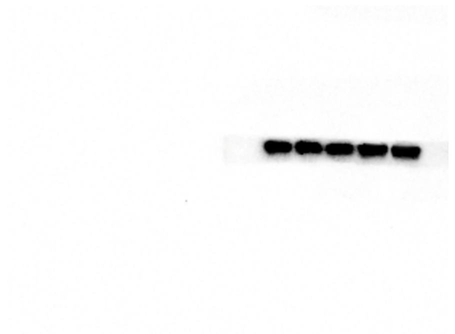

1

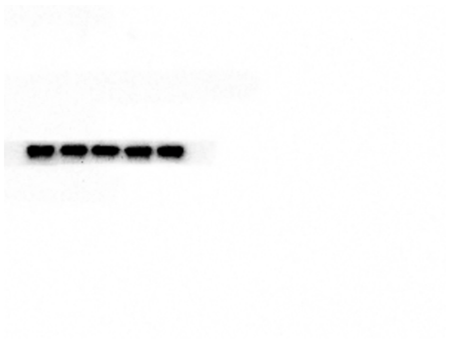

2

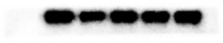

3

p-ERK

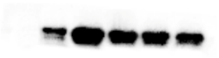

1

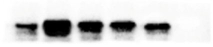

2

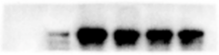

3

ERK

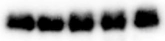

1

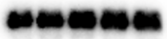

2

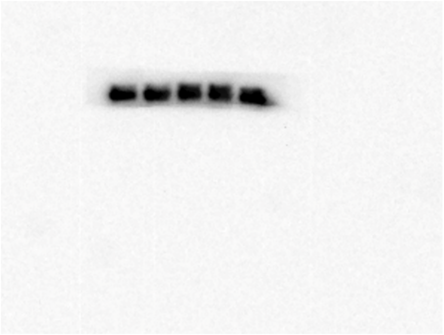

3

gp91phox

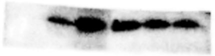

1

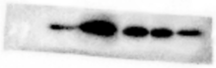

2

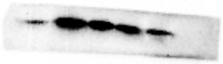

3

p22phox

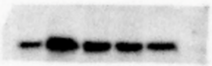

1

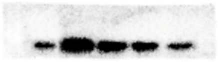

2

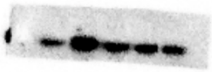

3

p47phox

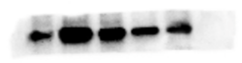

1

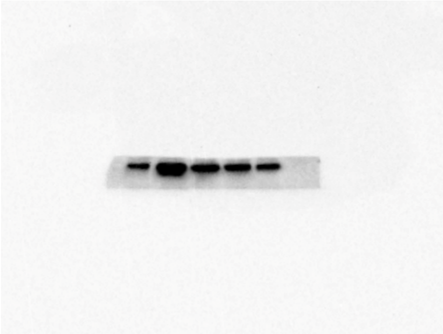

2

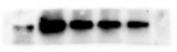

3

P67phox

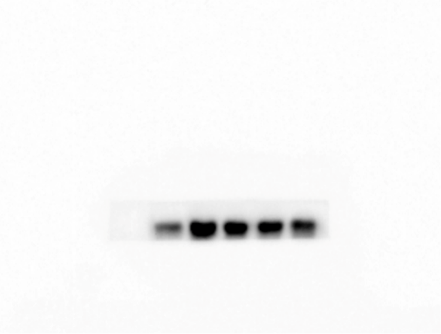

1

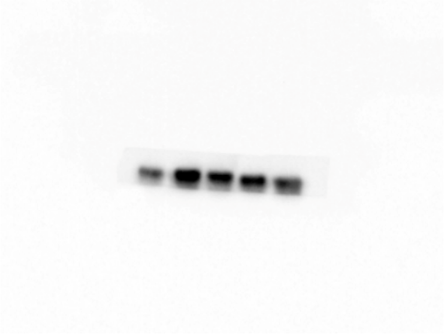

2

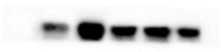

3

GAPDH

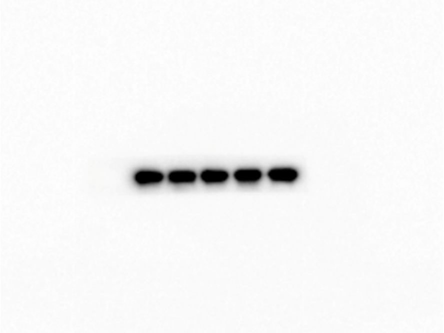

1

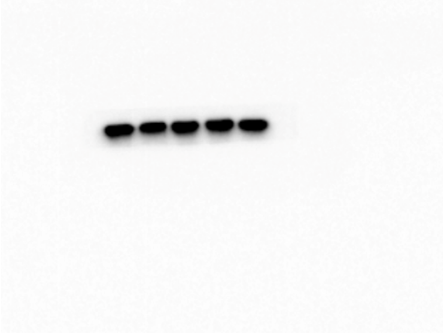

2

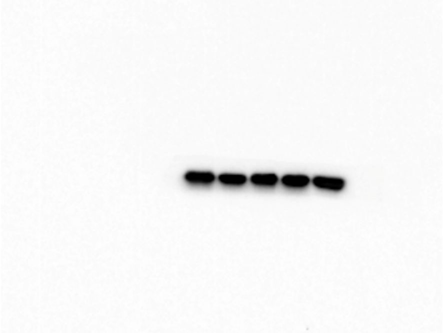

3
